# Dermatological manifestations of tick-borne viral infections found in the United States

**DOI:** 10.1186/s12985-022-01924-w

**Published:** 2022-11-28

**Authors:** Ammie Rupani, Hatem A. Elshabrawy, Jeremy Bechelli

**Affiliations:** 1 College of Osteopathic Medicine, Sam Houston State University, Conroe, TX 77304 USA; 2 Deparmtent of Molecular and Cellular Biology, College of Osteopathic Medicine, Sam Houston State University, Conroe, TX 77304 USA; 3 Department of Biological Sciences, College of Science and Engineering Technology, Sam Houston State University, Huntsville, TX 77340 USA

## Abstract

**Abstract:**

Tick-borne diseases (TBDs) are bacterial, viral, and parasitic diseases transmitted by ticks. Viral TBDs have increased in prevalence over the last decade with many new pathogenic viruses being discovered. Doxycycline is often empirically prescribed by clinicians to treat symptomatic patients following tick bites due to suspicions of bacterial TBDs such as Rocky Mountain spotted fever, anaplasmosis, and ehrlichiosis. However, viral TBDs are included in the differential diagnosis if patients do not clinically improve following antibiotic therapy. Several viral TBDs present with dermatological manifestations. Recognizing the differences in clinical presentations of TBDs, particularly of newly emerging viral TBDs in the United States, can help physicians identify the viral TBD, and possibly rule out viral illnesses with different clinical presentations. Therefore, this review discusses clinical manifestations, with an emphasis on dermatologic manifestations of Heartland Virus, Bourbon Virus, Powassan Virus, Deer Tick Virus and Colorado Tick Fever Virus.

**Key points:**

Viral tick-borne diseases have increased in prevalence over the last decade and often have similar clinical manifestations to other tick-borne diseases, including bacterial infections. Here, we review the dermatologic manifestations of Heartland Virus (HRTV), Bourbon Virus (BRBV), Powassan Virus (POWV), Deer Tick Virus (DTV) and Colorado Tick Fever Virus (CTFV) that are important for clinicians.

## Background

Tick-borne diseases (TBDs) have doubled in the United States in the last few years, necessitating a greater understanding of the signs and symptoms associated with TBDs for more accurate diagnoses and treatments. [1]. The increase in outdoor activities in parks and national forests during the COVID-19 pandemic combined with limited knowledge of precautionary steps to minimize tick bites, have resulted in a higher incidence of TBDs [2]. A recent study showed that in Delaware, where Lyme disease is prevalent, only 38.4% of people were aware of the TBD, and just 13.2% of the respondents changed their behavior to protect themselves from tick bites, suggesting a general lack of knowledge on the importance of preventing tick bites [3].

Ticks can transmit various pathogens, including bacteria, viruses, and parasites [4], including Lyme disease caused by *Borrelia burgdorferi*, spotted fever rickettsiosis (*Rickettsia rickettsii* and *R. parkeri*), Tularemia (*Francisella tularensis* ) and the parasitic agent *Babesia microti* [1]. Tick bites result in primary skin lesions and inflammation in the form of firm papules and intense pruritis, due to the immediate reactions to toxins and irritants in the tick saliva. However, tick bites may also develop into chronic edematous nodules due to inflammatory reactions to fragments from tick mouthparts. Moreover, more specific secondary lesions or dermatological presentations are dependent on the specific TBD transmitted, which is often helpful in differentiating between TBDs [5]. In this review, we discuss several emerging viral TBDs in the United States that are caused by Heartland Virus, Bourbon Virus, Powassan Virus, Deer Tick Virus, and Colorado Tick Fever Virus, including their dermatological manifestations.

## Heartland virus

Heartland Virus (HRTV) belongs to the genus *Phlebovirus*, family *Phenuiviridae*. HTRV’s genome is a negative single-stranded (-ss) RNA consisting of small, medium, and large segments. The small segment encodes for nucleocapsid protein and nonstructural proteins. The medium segment encodes for structural glycoproteins, Gn and Gc, and are targets of neutralizing antibodies, whereas the large segment encodes RNA-dependent RNA polymerase. Studies have shown that HRTV is genetically related to *Dabie bandavirus*, formally known as Severe Fever with Thrombocytopenia Syndrome Virus (SFTSV) or *Huaiyangshan Banyangvirus *[6].

HRTV was first identified in two Missouri farmers in 2009 [7]. The two farmers presented with fever, fatigue, anorexia, diarrhea, leukopenia, and thrombocytopenia, now known to be common presenting symptoms of patients with HRTV infection. HRTV-infected patients may also present a local rash at the site of the tick bite. However, case reports of HRTV have shown that patients typically do not have a rash at the site of tick bite, indicating that the rash is not reliable for the diagnosis of HRTV infections [6]. Complete blood counts of HRTV-infected patients show leukopenia and thrombocytopenia often combined with elevated transaminases, indicative of liver disfunction [6]. In addition, a study has reported a severe HRTV case with erythema on the left lower extremity showing 2 cm central necrosis[8]. Systemic viral dissemination can also cause additional symptoms such as altered mental status, gastrointestinal symptoms, and metabolic acidosis [8]. Molecular and serological testing of the HRTV is possible, but can only be performed at the CDC due to the lack of commercially available tests in the United States [9].

Since January 2021, more than 50 cases have been reported in the Midwestern and Southern United States; specifically in Arkansas, Georgia, Illinois, Indiana, Iowa, Kansas, Kentucky, Missouri, North Carolina, Oklahoma, and Tennessee [10]. Most cases occur in the summer months, typically 2 weeks post tick sighting on patients. The geographical distribution of cases closely matches the distribution of the lone star tick, *Amblyomma americanum*, which was later confirmed as the primary vector of HRTV [11, 12]. Clinical signs and symptoms of HRTV infections overlap with the more prevalent disease, human monocytotropic ehrlichiosis (HME), which was first discovered in 1987. *Ehrlichia chaffeensis* is the bacterial causative agent of HME and is also transmitted by the Lone Star tick in the Midwest region of the United States [13]. Symptoms of HME range from mild febrile illness to multi-organ failure. Unlike HRTV, HME has a more distinctive rash that appears as a maculopapular, petechial, or diffuse erythema that affects the whole body except the face, palms, and soles of the feet [14]. Due to the efficacy of doxycycline in treatment of HME, it is recommended to start patients on doxycycline, then evaluate the patients for the possibility of HRTV if symptoms do not resolve. There are currently no vaccines or antiviral drug treatments for HRTV.

## Bourbon virus

Bourbon virus (BRBV) belongs to the genus *Thogotovirus*, family *Orthomyxoviridae*. BRBV is a novel enveloped negative-sense RNA virus and consists of six segments in its genome that are predicted to encode for PB2, PB1, and PA polymerase proteins, a nucleoprotein (NP), a surface glycoprotein (GP), and a matrix (M) protein [15]. A single glycoprotein embedded in the viral envelope mediates entry into host cells [16]. Like HRTV, the lone star tick (*Amblyomma americanum*) is the vector of BRBV [17, 18].

BRBV was first identified when a patient from Bourbon County, Kansas, died in 2014 after multiple tick bites. Since the discovery of the Bourbon virus, the virus has been a relatively rare tick-borne viral illness in the United States, with the CDC reporting only a few cases [19]. The cases have been identified mainly in the Midwest and Southern United States. However, there is some overlap with the HRTV in Kansas [19].

It has been reported that BRBV-infected individuals develop fever, anorexia, nausea, vomiting, myalgia, and arthralgia. In addition, they may present with diffuse maculopapular or a papular rash that appears on the torso [20]. Blood tests show leukopenia, lymphopenia, thrombocytopenia, hyponatremia, and increased aminotransferases [20]. It has also been reported that progressive BRBV infections could lead to petechiae, which has been noted on the lower extremities and soft palate of onepatient [20].

Similarly, the Dhori virus (DHOV) is transmitted by metastriate ticks (non-Ixodes ticks) and mosquitoes. DHOV is found in Europe, North Africa, and western and central Asia and share 70% genome sequence identity with BRBV in multiple genomic segments [20]. Unlike BRBV infections, DHOV infections are characterized by encephalitis in 40% of cases including headache and retrobulbar pain [17]. There are no reported dermatological manifestations of DHOV, which could distinguish DHOV infections from HRTV and BRBV infections, further highlighting the importance of recognizing dermatological and clinical manifestations to allow differentiation between viral TBDs.

Currently, there are no routine laboratory tests that can confirm the diagnosis of BRBV infections. Only supportive care (including anti-pyretic, analgesics, and I.V. fluids) can be offered to the BRBV-infected patients since there are currently no vaccines or antiviral treatment approved for BRBV [21].

## Powassan virus and deer tick virus

Powassan virus (POWV) is the only North American member of the family *Flaviviridae* (+ ss RNA viruses), genus *Flavivirus*, that is transmitted by the *Ixodes* tick species, causing tick-borne encephalitis. The POWV genome comprises seven genes coding for nonstructural proteins and three genes coding for structural proteins, including the capsid and envelope proteins [22]. Recently, two genetic lineages of POWV have been described; lineage I was named POWV, whereas lineage II was renamed as Deer Tick Virus (DTV; discussed in the next section) [22]. POWV infects macrophages and dendritic cells, which then transport the virus to the lymphatic system leading to systemic viral dissemination [23].

The virus was first identified in 1958 and named after a 5-year-old child who died from encephalitis in Powassan, Canada [24]. POWV infections have increased from one case per year prior to 2005 to 10 cases per year after 2005 [25]. Between 2011 and 2020, 194 documented cases of Powassan virus disease cases had been reported in the USA [26]. POWV cases are primarily reported during the season of high tick activity (May-September), in the north-central and northeastern states of the United States. Between 2010 and 2019, 181 cases were reported in the United States, with 166 cases developing the neuroinvasive disease. Human PWOV infections have also been reported in Canada and Russia [26].

POWV infections can result in neuroinvasive or non-neuroinvasive diseases. Patients with non-neuroinvasive disease present with sore throat, drowsiness, headache, disorientation, and rarely fever. However, POWV infections are primarily neuroinvasive, a distinctive feature compared to other tick-borne illnesses [27]. The neuroinvasive signs of POWV infections include encephalitis, meningoencephalitis, and aseptic meningitis. Ophthalmoplegia and direction-changing nystagmus have also been reported in some cases of POWV encephalitis. Death is reported in 10% of neuroinvasive cases, whereas 50% of surviving patients report long-term neurological sequelae including hemiplegia, muscle wasting, acute headaches, and memory problems [22].

A faint, diffuse maculopapular rash on the trunk has been described in a few POWV-infected patients [28], which can include the extremities and back, but is not associated with the tick bite area. A study showed that only three of eight POWV patients presented with rash [28]; this indicates that rash is not a common presentation in POWV infections. However, it can still be used with other symptoms and diagnostic tests to diagnose POWV infection [28–30]. Laboratory diagnosis of POWV infection involves detecting IgM antibodies using enzyme-linked immunosorbent assay (ELISA) and confirmation using plaque neutralization assay. A four-fold increase in antibody titer or IgM detection in CSF is diagnostic of POWV infections [28]. Like HRTV and BRBV, there are no vaccines or definitive antiviral treatments for POWV. There are some documented cases of successful treatment with high-dose corticosteroids and IVIG. However, neither are approved treatments for POWV [28].

As mentioned earlier, DTV is a genetic variant (lineage II) of POWV. They share 84% and 94% genomic sequence identity and amino acid sequence identity, respectively, and cannot be distinguished serologically [31]. DTV is transmitted by the Rocky Mountain wood tick, *Dermacentor andersoni*, but is mainly found in deer ticks (*I. scapularis*) collected from the northeastern United States. Three cases of DTV infections have been reported in the literature through 2017 [32].

DTV can cause severe central nervous system infections in humans, similar to POWV. Symptoms include fever, arthralgias, and headache. Laboratory tests of DTV-infected patients show thrombocytopenia with possible acute kidney injury [33]. Unlike POWV, case reports of DTV infections have shown erythema migrans rash near the site of the tick bite [33]. Erythema migrans rash is also associated with Lyme disease, which is a common bacterial tick-borne disease. The critical distinguishing feature between the two illnesses is the presence of encephalitis, and bilateral maculopapular palmar rash, more commonly found during DTV infection [34, 35]. DTV can be confirmed by serological testing if suspected in a patient. Currently, there are no treatments or vaccines available, and only supportive treatment can be provided for DTV infections [36].

## Colorado tick fever virus

Colorado Tick Fever Virus (CTFV) is a member of the genus *Coltivirus*, family *Reoviridae *[37]. Viruses within the genus *Coltivirus* have double-stranded RNA genomes made of 12 segments that encode for 13 viral proteins (VP) 1–12 [38]. In the life cycle of CTFV, the virus is primarily maintained in nature by *Dermacentor andersoni* ticks. In contrast, the main vertebrate reservoir is *Spermophilus lateralis* (golden-mantled ground squirrel) and several other mammals, including chipmunks, wild mice, wood rats, wild rabbits, deer, elk, sheep, and coyotes [39].

CTFV infections are the second most reported arboviral infections in the United States after West Nile virus infections, with approximately 200–400 cases reported annually [40, 41]. CTFV cases have been reported in the western United States in California, Colorado, Idaho, Montana, Nevada, New Mexico, Oregon, South Dakota, Utah, Washington, and Wyoming. However, the risk of acquiring CTFV increases at elevations above 7000 feet [42].

Typically, CTFV-infected patients present with biphasic fever, headache, myalgia, and fatigue. In addition, laboratory tests show leukopenia and thrombocytopenia. CTFV-infected patients could develop severe complications such as meningitis, encephalitis, and bleeding disorders, and around 20% of patients require hospitalization [43]. However, fatalities due to CTFV infections are rare [44].

Macular, maculopapular, and petechial rash, together with hyperesthetic skin, can be associated with CTFV infections in 5–15% of cases [45, 46]. A recent paper attributed the rash to dermal microvascular endothelial cells which are susceptible to CTFV infection and undergo apoptosis [47]. Palatal enanthem, small spots on the mucous membrane, can also be seen as a dermatological manifestation of CTFV infections [40, 44, 48, 49].

Similar to CTFV, Eyach virus (EYAV) which shares 55–88% genetic sequence identity with CTFV presents with a febrile illness [50]. However, dermatologic manifestations are uncommon. Moreover, EYAV causes neurologic complications in a higher number of patients than CTFV; therefore, the presentation of a rash may help distinguish between CTFV and Eyach viral infections, especially if the patient recently traveled to Germany or France, where EYAV has been found in ticks [43]. In addition, Salmon River virus causes a similar disease as CTFV [51]. However, the pathogenicity of California Hare Coltivirus (CTFV-Ca) is currently unknown. Therefore, distinguishing between CTFV and related coltiviruses from EYAV is heavily reliant on serological and molecular testing [50].

CTFV infections can be diagnosed using serologic tests to detect anti-coltivirus antibodies. These tests include complement fixation tests, seroneutralization assays, immunofluorescence assay, ELISA, and Western immunoblotting. However, serology may not be timely since antibody production can take 14–21 days. More commonly, real-time reverse transcriptase-polymerase chain reaction (RT-PCR) assays are used to detect CTFV RNA or the RNA of its cross-reacting serotypes, including CTFV-Ca and Salmon River virus. Intracerebral inoculation of infected human blood into suckling mice can also be used in the isolation and subsequent diagnosis of coltiviruses, though this method is not practical for most laboratories and clinicians. There are no FDA-approved vaccines or antiviral treatment options for CTFV infections, and only supportive care based on the patient’s symptoms is recommended [52].

## Conclusion

“One world, one health” is a call for an interdisciplinary approach to science due to the interconnected nature of human actions, animals, and ecological health. The rising number of tick-borne diseases and the newly emerging diseases, such as HRTV and Bourbon virus, is an incentive to manage these diseases with an interdisciplinary approach, especially between practitioners and virologists. This article discusses a multidisciplinary approach by bridging viral knowledge with clinical knowledge to aid clinicians in recognizing presentations of tick-borne diseases.

Tick-borne diseases of bacterial origin present with distinctive dermatological manifestations, such as erythema migrans in Lyme disease, caused by *Borrelia burgdorferi*, and the petechial rash in Rocky Mountain Spotted Fever, caused by *Rickettsia rickettsii* [53, 54]. Additionally, Southern tick-associated rash illness (STARI) is an emerging zoonotic disease spread by *A. americanum*, that presents with an annular rash that is almost identical to erythema migrans with lymphocytic dermal infiltrate seen in Lyme disease [55]. While STARI is believed to be caused by *Borrelia lonestari*, there is some debate regarding its exact etiology [56–58].

TBDs of bacterial origin are studied more due to the prevalence in the United States, and as stages of the disease with distinct clinical symptoms have been identified. As a result, bacterial tick-borne infections are also stressed in medical schools across the United States and competency exams (USMLE and COMLEX). In contrast, TBDs of viral origin, primarily those recently discovered, have not been studied as extensively, and more research is needed to distinguish characteristic symptoms, like cutaneous presentations. Underreporting and underdiagnosis of viral tick-borne illness can occur due to overlapping dermatological indications of viral and bacterial tick-borne disease. Many viral TBDs can be mistaken for bacterial TBDs without adequate serological testing.

Bacterial TBDs and viral TBDs can also often overlap due to ticks harboring multiple diseases. For example, the *Ixodes scapularis* tick that carries Lyme disease can also cause co-infections with Powassan virus [1]. Co-infection has also been shown to enhance disease severity, or alter typical symptoms, thus impeding diagnosis. Lyme disease patients with co-infections presented with more influenza-like symptoms than those with Lyme disease alone [59]. Co-infection can make recognition of dermatological manifestations of tick-borne diseases more important as they can aid clinicians in deciding diagnostic testing to order and treatment plan.

Cutaneous presentations of these viral tick-borne illnesses inform practitioners and patients about which viral disease was transmitted from the tick. The knowledge of the viral particle guides treatment and can prevent death. Performing a detailed skin exam on a patient presenting post tick bite or with symptoms after visiting an area endemic with these diseases helps identify cutaneous presentations of illnesses, such as maculopapular rash, erythema migrans, or petechial rash. Further research on the treatment of tick-borne viral diseases is needed to prevent deaths, mainly due to the doubling in tick-borne disease cases in the past ten years in the United States.

**Fig. 1:**
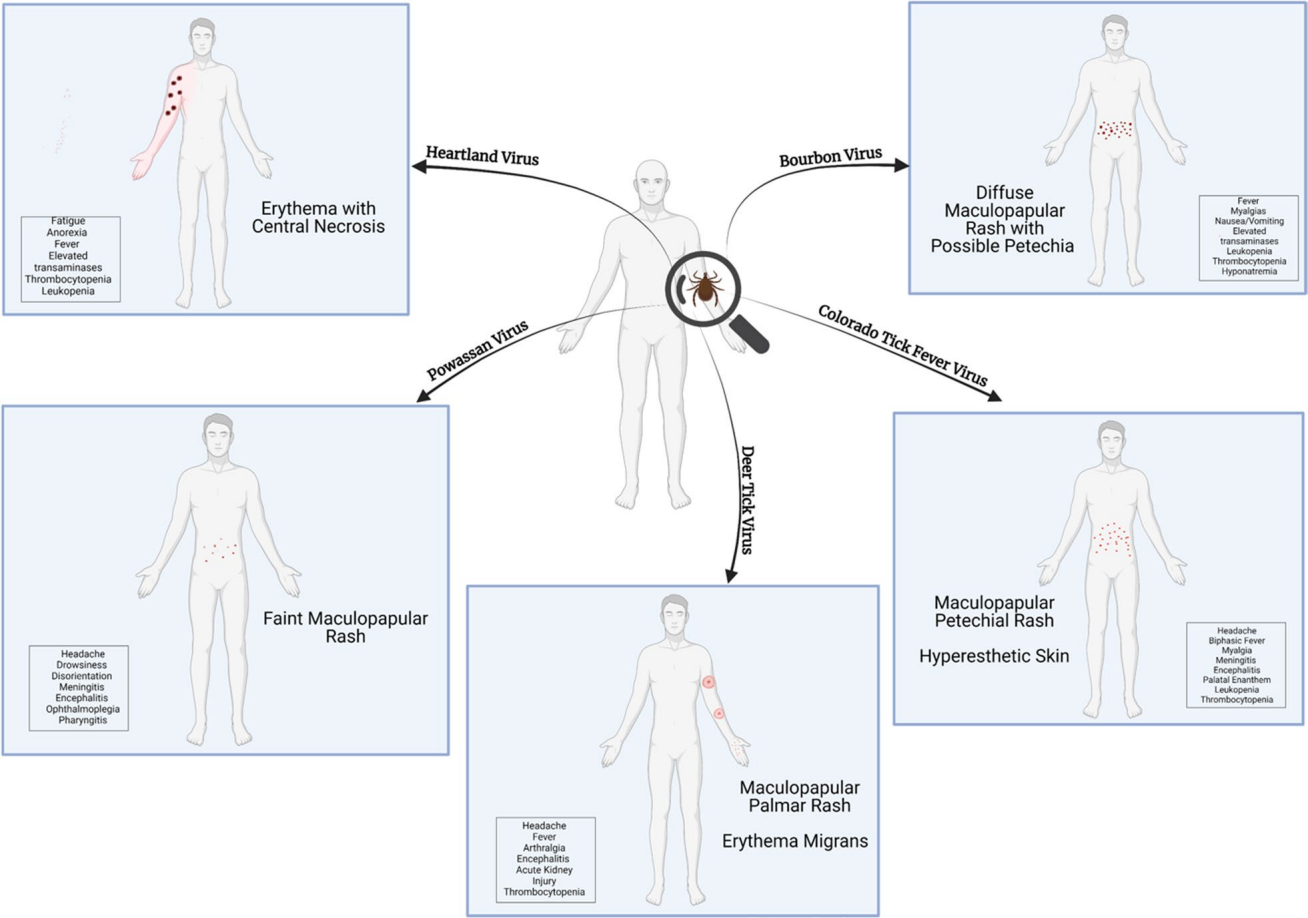
The key symptoms of each tick-borne illness are illustrated in the figure above. Infection with Heartland virus (HRTV) presents with fever, anorexia, and fatigue. Cases have shown an erythematous rash with central necrosis in HRTV-infected patients; however, the rash is not a reliable symptom for diagnosis. Laboratory findings will include thrombocytopenia, leukopenia, and elevated transaminases (aspartate transaminase and alanine transaminase). Bourbon virus-infected individuals develop fever, anorexia, nausea, vomiting, myalgia, and arthralgia. In addition, they may present with diffuse maculopapular or a papular rash that appears on the torso. Laboratory findings will show leukopenia, lymphopenia, thrombocytopenia, hyponatremia, and increased transaminases. Patients with Powassan virus (POWV) infections present with neuroinvasive or non-neuroinvasive diseases. Non-neuroinvasive POWV condition presents with a sore throat, drowsiness, headache, disorientation, faint maculopapular rash, and rarely fever. Neuroinvasive presentations of POWV infections include encephalitis, meningoencephalitis, ophthalmoplegia, and aseptic meningitis. Deer Tick virus, a genetic variant of POWV, presents with similar symptoms, including the distinctive erythema migrans rash, bilateral palmar rash, and possible progression to acute kidney injury. Colorado Tick Fever virus-infected patients present biphasic fever, headache, myalgia, maculopapular rash, and fatigue. Patients may also uniquely present with hyperesthesia or skin which is highly sensitive to stimulation. Laboratory tests show leukopenia and thrombocytopenia. Each of the five viral tick-borne illnesses presents unique clinical symptoms, and recognizing them is key to diagnosing and treating the patient

There are no specific drug therapies, or FDA-approved vaccines for tick-borne viral infections in the United States. While hospitalization is common for these infections, treatment is mainly supportive. Personal protection, landscape management, and wildlife management are effective for preventing and controlling tick-borne viral infectious diseases, but vaccines and drug therapies are critically needed. A convenient area to report a case of tick-borne disease is also required, especially because patients with mild forms of TBDs do not come to the hospital, and those mild symptoms are not documented. A website through the CDC where patients can add their symptoms and their location can aid in solving the underreporting of tick-borne illnesses while giving insights on mild forms of the diseases. The database can also lead to more distinctive dermatological manifestations by stage of the disease (Fig. 1, Table 1).

**Table 1:** The key dermatological symptoms of each tick-borne illness

Virus	Reported Dermatological Manifestations	References
Heartland Virus (HRTV)	Erythema with central necrosis	[6–8]
Bourbon Virus (BRBV)	Diffuse maculopapular or papular rash on torso, possible progression to petechia	[20]
Powassan Virus (POWV)	Faint, diffuse maculopapular rash on trunk	[28]
Deer Tick Virus (DTV)	Erythema migrans rash	[33]
Colorado Tick Fever Virus (CTFV)	Macular, maculopapular, and petechial rash together with hyperesthetic skin	[40, 44–49]

## Data Availability

Not applicable.
